# C.N.A. Nurses and the "Roll of Merit"

**Published:** 1917-09-29

**Authors:** A. M. T. Millington

**Affiliations:** "Moorside Cottage," Moordown, Bournemouth


### THE EDITOR'S LETTER-BOX.

### C.N.A. Nurses and the "Roll of Merit."

*To the Editor of* THE HOSPITAL.

SIR,—My attention has been called to a paragraph in your valuable paper of August 18, 1917 (page 403, Report of Colonial Nursing Association), which evidently refers to myself under the name of Nurse Ann L. F. Milligan; it should be Nurse Ann M. T. Millington.

I believe the "Roll of Merit" is the outcome of a suggestion of mine that when, after long years of active service, we resigned and our names were taken off the books, might we not be kept in remembrance in a separate register. This was brought before the committee, and the "Roll of Merit" was the result.—I am, Sir, yours truly, A. M. T. MILLINGTON.

"Moorside Cottage," Moordown, Bournemouth,
September 20, 1917.

